# Nitrogen partitioning and microbial protein synthesis in lactating dairy cows with different phenotypic residual feed intake

**DOI:** 10.1186/s40104-019-0356-3

**Published:** 2019-06-14

**Authors:** Yunyi Xie, Zezhong Wu, Diming Wang, Jianxin Liu

**Affiliations:** Institute of Dairy Science, MOE Key Laboratory of Molecular Animal Nutrition, College of Animal Sciences, Zhejiang University, Hangzhou, 310058 People’s Republic of China

**Keywords:** Lactating cows, Microbial protein, Nitrogen partitioning, Residual feed intake

## Abstract

**Background:**

Residual feed intake (RFI) is an inheritable measure of feed efficiency that is independent on level of production. However, physiological and metabolic mechanisms underlying divergent RFI are not fully elucidated. This study was conducted to investigate dietary nitrogen (N) partitioning and microbial protein synthesis in lactating dairy cows divergent in phenotypic RFI.

**Results:**

Thirty Holstein dairy cows (milk yield = 35.3 ± 4.71 kg/d; milk protein yield = 1.18 ± 0.13 kg/d; mean ± standard deviation) were selected for the experiment to derive RFI. After the RFI measurement period of 50 d, the 10 lowest RFI cows and 8 highest RFI cows were selected. The low RFI cows had lower dry matter intake (DMI, *P* < 0.05) than the high RFI cows, but they produced similar energy-corrected milk. The ratios of milk to DMI (1.41 vs. 1.24, *P* < 0.01) and energy-corrected milk to DMI (1.48 vs. 1.36, *P* < 0.01) were greater in low RFI cows than those in the high RFI cows. The low RFI cows had lower milk urea nitrogen than that in the high RFI cows (*P* = 0.05). Apparent digestibility of nutrients did not differ between two groups (*P* > 0.10). Compared with high RFI animals, the low RFI cows had a lower retention of N (5.72 vs. 51.4 g/d, *P* < 0.05) and a higher partition of feed N to milk N (29.7% vs. 26.5%, *P* < 0.05).

**Conclusions:**

The results suggest that differences in N partition, synthesis of microbial protein, and utilization of metabolizable protein could be part of the mechanisms associated with variance in the RFI.

**Electronic supplementary material:**

The online version of this article (10.1186/s40104-019-0356-3) contains supplementary material, which is available to authorized users.

## Background

The optimization of milk production (especially milk protein) per kilogram of feed consumed is important to dairy farmers [1]. Residual feed intake (RFI) is a measure of feed efficiency and defined as the difference between the expected and actual feed intake to support maintenance and production over a specific production period [2]. There is growing evidence that feed efficiency is different even in high-efficiency herds that feed the same diet [3]. The low RFI (efficient) cows are those that consume less feed than the expected for maintenance and production, compared with the high RFI cows, and RFI is a heritable trait for dairy cows [4–6]. Several mechanisms have been suggested to explain the causes of variation in RFI between dairy cows, including rumen microbial populations [7], feeding behavior [8, 9] and nutrients digestibility [10, 11]. Understanding the mechanisms for different RFI in dairy cows may help dairy industry make informed breeding decisions.

Variation in the RFI of lactating cows may be related to nitrogen (N) partitioning. It is reported that apparent N digestibility is greater for lower RFI animals [10, 12]. Furthermore, the RFI variation in cattle could be explained by variation in rumen-related functions such as feed degradation and microbial protein (MCP) synthesis, suggesting the vital role of rumen for divergence in RFI. Microbial protein has a major impact on the quantity and quality of the metabolizable protein (MP) that is delivered to and absorbed from the small intestines [13]. The improvement of efficiency of MCP synthesis is important because it allows dairy cows to optimize the protein available to the animals.

Milk protein yield is crucial milk performance trait of dairy cows that directly affect the dairy profits. Thus, we hypothesized that individual variation exists for RFI in the relatively higher milk-protein-yielding cows. In these cows, low RFI animals would produce MCP and use MP more efficiently than the high RFI cows. Thus, the objective of this study was to identify which processes, from intake to milk protein secretion, contribute to the differences in the RFI.

## Materials and methods

### Animals and management

All experimental procedures involving animals were approved by the Animal Care Committee of Zhejiang University (Hangzhou, China). Thirty lactating Holstein cows (BW = 749 ± 74.6 kg, days-in-milk = 189 ± 18.9 d; mean ± standard derivation) were selected for the experiment. These cows had relatively higher milk yield (35.9 ± 4.20 kg/d) and milk protein content (3.29% ± 0.22%). The trial lasted for 57 d, with the first 7 d used for adaptation. The cows were fed total mixed ration that was formulated to produce 35 kg of milk a day with 3.25% of milk protein based on the NRC recommendation [13]*,* and the ration ingredient is shown in Table 1. Feed intake data was collected using automatic weighing troughs (Roughage Intake Control System, Marknesse, The Netherlands). Each feeding station included an individual identification system that allowed each cow to enter a specific feeding bunk and automatically recorded each meal. The cows were milked three times daily at 06:30, 14:00 and 21:30 h, fed three times daily at 07:00, 14:30 and 22:00 h to enable *ad libitum* intake with 5% to 10% refusal, and had free access to drinking water. The feed residual was discarded daily before the morning feeding.

**Table 1:** Ingredient composition of the total mixed rations

Ingredient	% (DM basis)	Nutrient levels^a^	% of DM
Alfalfa hay	16.1	DM	51.8
Oat hay	7.55	OM	95.5
Corn silage	18.8	CP	16.0
Brewer’s grains	3.47	NDF	32.9
Beet pulp	4.67	ADF	19.0
Cottonseed meal, whole	5.49	NE_L_, Mcal/kg DM	1.70
Steam-flaked corn	5.57		
Ground corn grain	17.0		
Soybean meal	9.62		
Expanded soybean	2.74		
Fat meal	1.09		
DDGS^b^	4.62		
CaHPO_4_	0.11		
NaCl	0.21		
Limestone	0.36		
NaHCO_3_	0.34		
MgO	0.13		
Premix^c^	2.12		

^a^*DM* dry matter, *OM* organic matter, *CP* crude protein, *NDF* neutral detergent fiber, *ADF* acid detergent fiber, *NE*_*L*_, net energy for lactation, estimated according to the NRC recommendation [13]

^b^*DGGS* Distillers Dried Grains with Solubles

^c^Premix, formulated to provide (per kg of DM): vitamin A ≥ 600 kIU, vitamin D_3_ ≥ 150 kIU, vitamin E ≥ 2,000 IU, nicotinic acid ≥500 mg, Cu ≥ 1500 mg, Fe ≥ 1,500 mg, Mn ≥ 1,500 mg, Zn ≥ 7,000 mg, I ≥ 90 mg, Se ≥ 50 mg, Co ≥ 20 mg

### Sample collection and measurements

Samples of the ration were collected weekly, and spot fecal samples (approximately 500 g) were collected from the rectum of each cow 3 times per day at 06:00, 12:00, and 18:00 h on d 24–25 and d 49–50 of the feeding period. The samples were dried at 65 °C for 48 h and then ground through a 1-mm screen in a Cyclotec mill (Tecator 1093; Tecator AB, Hoganas, Sweden) for later analysis. All the samples were analyzed for dry matter (DM, method No. 934.01), crude protein (CP, method No. 988.05), crude ash (method No. 942.05), and acid detergent fiber (ADF, method No. 973.18) according to AOAC methods [14]. The content of neutral detergent fiber (NDF) content was analyzed by the method of Van Soest et al. [15]. Chemical composition of the diet is listed in Table 1.

The *in situ* rumen DM and CP degradation of diet sample was determined through the ruminal incubation of samples for 2, 4, 8, 12, 16, 24, 36, and 48 h according to the method described by Wang et al. [16]. The residues and original diet samples were analyzed for DM and CP. The DM and CP degradation contents are listed in Additional file 1: Table S1. Indigestible NDF (iNDF, 12-day ruminal incubation in 25-μm-pore-size bags) was used as an intrinsic marker to estimate fecal excretion and nutrient digestibility and was determined according to the methods of Lee and Hristov [17].

Milk yields from all cows were recorded at each milking. Milk samples were collected weekly for consecutive two days at a proportion of 4:3:3 according to three times of milking by using composite milk samplers. Bronopol tablets (milk preservative, D & F Control Systems, San Ramon, CA) were added to the composite milk samples, which were stored at 4 °C for future analysis of protein, fat, lactose, milk urea nitrogen (MUN), total solids (TS), and somatic cell counts (SCC) using a spectrophotometer (Foss-4000; Foss Electric A/S, Hillerød, Denmark).

On d 24 and 49 of the feeding period, rumen fluid (50 mL) was collected using an oral stomach tube approximately 3 h after the morning feeding, as described by Shen et al. [18]. The pH of the rumen fluid was immediately measured using a portable pH meter (FE20-FiveEasy Plus™; Mettler Toledo Instruments Co. Ltd., Shanghai, China). The samples were placed on ice and kept stationary while the supernatant separated, and then, the samples were frozened at − 20 °C for future determination of volatile fatty acid (VFA). Two mL of rumen sample was acidified with 20 μL of 25% orthophosphate acid and then centrifuged at 20,000×*g* for 10 min at 4 °C. The supernatant was then subjected to VFA measurement using a gas chromatograph (GC-2010, Shimadzu, Kyoto, Japan) according to the methods described previously [19].

Blood samples were obtained from the coccygeal vein of each cow into procoagulation 10 mL tubes approximately 3 h after feeding on d 24 and d 49 of experimental period. The samples were then centrifuged at 3,000×*g* for 15 min to collect serum and were frozen at − 20 °C until analysis. Serum samples were analyzed using an autoanalyzer 7020 (Hitachi High-Technologies Corp., Tokyo, Japan) for total plasma protein, albumin, globulin, urea nitrogen, creatinine, glucose, nonesterified fatty acids (NEFA), triglycerides, cholesterol, alkaline phosphatase (ALP), aspartate aminotransferase (AST), alanine aminotransferase (ALT) and total bilirubin, according to the method described by Richardson et al. [20].

Cows were weighed weekly immediately before the morning feeding. The average daily gain (ADG) was calculated based on the difference between the BW on the first day and the last day during the experimental period.

### Calculation of MCP and MP

Urinary purine derivatives (PD) were used to indirectly estimate the MCP flow in the rumen [21]. Spot urine samples were collected on d 24–25 and d 49–50, i.e., d 24 and 49, 08:00, 16:00 and 24:00 h; d 25 and 50, 12:00, 20:00 and 04:00 h. Collected urine samples were pooled by cow, and 15 mL of each subsample was acidified with 60 mL of 0.036 mol/L H_2_SO_4_ and immediately stored at − 20 °C for later analysis of the PD [21]. Creatinine was analyzed using a colorimetric picric acid assay [22]. Creatinine has been validated as a marker to estimate urine volume [23] and was assumed to be excreted at a rate of 29 mg/kg of BW [24]. The intestinally absorbable dietary protein (IADP) was estimated by the following equation: IADP = RUP × CP intake × IDP, where IDP is the intestinal digestibility of rumen undegraded protein (RUP), which was determined from the residue of feedstuff incubated in the rumen for 16 h, according to a modified 3-step procedure [25].

### RFI computation

The expected feed intake was calculated based on methods developed for lactating dairy cattle [6]. Stepwise multiple linear regression analysis was used to establish the regression equation:

$$ {Y}_{\mathrm{i}}={\beta}_0+{\beta}_1{\mathrm{ECM}}_{\mathrm{i}}+{\beta}_2{{\mathrm{BW}}^{0.75}}_{\mathrm{i}}+{\beta}_3\mathrm{ADG}+{e}_i, $$

where *Y*_i_ is the expected feed intake (kg/d) of the *i*th animal, *β*_0_ is the regression intercept, *β*_1_ is the partial regression coefficient for energy-corrected milk yield (ECM, kg/d), *β*_2_ is the partial regression coefficient for metabolic BW (BW^0.75^, kg), *β*_3_ is the partial regression coefficient for ADG (kg), and *e*_*i*_ is the random error associated with the *i*^th^ animal.

The RFI for each animal was calculated as the difference between the actual and expected average feed intakes during the trial. Cows with RFI > 0.3 SD above the mean of 0 were categorized as the “low-efficiency group” and defined as high RFI; those with RFI > 0.3 SD below the mean were categorized as the “high-efficiency group” and defined as low RFI. Thus, there were 10 and 8 cows in low- and high-RFI groups, respectively. The days-in-milk, parity and body weight of the cows for two groups are shown in Table 2.

**Table 2:** Productivity of lactating cows selected for phenotypic divergence in residual feed intake (RFI)

Items	Low RFI	High RFI	SEM	*P* value^a^		
				RFI	Day	RFI × Day
Number, head	10	8				
Lactation performance^b^						
DMI, kg/d	24.2	26.6	0.68	0.02	< 0.01	0.32
Milk yield, kg/d	34.0	33.1	1.39	0.65	< 0.01	0.96
Milk protein yield, kg/d	1.11	1.10	0.05	0.90	0.03	0.91
ECM, kg/d	35.7	36.4	1.35	0.72	< 0.01	0.85
ECM/DMI, kg/kg	1.48	1.36	0.03	< 0.01	< 0.01	0.77
Milk/DMI, kg/kg	1.41	1.24	0.04	< 0.01	< 0.01	0.64
RFI, kg/d	−0.96	1.18	0.22	< 0.01	–	–
Milk composition^c^						
Milk fat, %	3.83	4.23	0.12	0.01	0.05	0.87
Milk protein, %	3.26	3.33	0.06	0.40	< 0.01	0.64
Lactose, %	4.97	4.90	0.03	0.21	< 0.01	0.15
Total solids, %	12.7	13.0	0.14	0.12	0.12	0.90
MUN, mg/dL	12.7	13.9	0.39	0.05	< 0.01	0.62
SCC, × 10^3^/mL	50.5	38.7	9.46	0.39	0.33	0.46
Days in milk	193	182	6.30	0.22	–	–
Parity	2.70	2.50	0.22	0.56	–	–
Body weight, kg	739	737	16.7	0.96	< 0.01	0.24
Average dairy gain, kg	0.17	0.26	0.18	0.66	0.08	0.68

^a^*P* value associated with RFI, time, and the interaction of RFI and time

^b^DMI, dry matter intake; ECM (kg) = 0.3246 × milk yield (kg) + 13.86 × milk fat (kg) + 7.04 × milk protein (kg) [44]. All cows remained pregnant during the trial

^c^*MUN* milk urea nitrogen, *SCC* somatic cell counts

### Statistical analysis

Data on lactation performance, digestibility, N conversion, urinary PD, and rumen fermentation variables were analyzed using PROC MIXED of SAS (SAS Institute, 2000). The model included the fixed effects of the RFI group (high and low), week, group × week, and random effects of the cow. The statistical significance was declared at *P* ≤ 0.05 and trends were indicated at 0.05 < *P* ≤ 0.10.

## Results

### Milk production

Milk yield, milk composition, BW and ADG of the lactating cows are summarized in Table 2. No differences were observed in the BW (*P* = 0.96) and ADG (*P* = 0.66) between higher and low RFI cows. The low RFI cows consumed 2.45 kg DM/d less than the high RFI animals (*P* = 0.02), but cows in both groups produced similar milk yield (*P* = 0.65), milk protein yield (*P* = 0.90) and ECM (*P* = 0.72). Low RFI cows had a lower content of milk fat (*P* = 0.01) and MUN (*P* = 0.05) than that of high RFI cows. The ratios of milk to DMI and ECM to DMI were greater in the low RFI cows than those in the high RFI cows (*P* < 0.01). The day of sampling had a significant (*P* < 0.05) effect on the milk yield and milk composition except for the total solids (*P* = 0.12) and SCC (*P* = 0.33).

### Rumen fermentation parameters and apparent digestibility

The rumen fermentation parameters and apparent digestibility of the lactating cows are listed in Table 3. The concentration of propionate was lower in low RFI group compared with that in the high RFI ones (*P* = 0.04, data not shown). However, there was no difference in the rumen pH (*P* = 0.35), total VFA (*P* = 0.18), the molar proportions of individual VFA (*P* > 0.10) and apparent digestibility (*P* > 0.10) between the two groups.

**Table 3:** Rumen pH, volatile fatty acids (VFA), and apparent digestibility of lactating cows selected for phenotypic divergence in residual feed intake (RFI)

Items	Low RFI	High RFI	SEM	*P* value^a^		
				RFI	Day	RFI × Day
pH	6.41	6.30	0.08	0.35	0.54	0.86
Total VFA, mmol/L	101	108	3.84	0.18	0.21	0.22
Molar proportion, mmol/100 mmol						
Acetate (A)	65.1	64.2	0.86	0.46	0.28	0.61
Propionate (P)	20.2	21.5	0.94	0.36	0.08	0.40
Butyrate	11.1	10.8	0.39	0.71	0.17	0.87
Isobutyrate	0.72	0.68	0.04	0.44	< 0.01	0.43
Valerate	1.45	1.45	0.05	0.98	< 0.01	0.30
Isovalerate	1.45	1.30	0.08	0.21	0.06	0.71
A: P ratio	3.29	3.11	0.18	0.46	0.07	0.40
Apparent digestibility^b^						
DM, %	64.0	64.4	0.87	0.73	0.12	0.43
CP, %	65.4	66.2	1.55	0.73	0.24	0.69
NDF, %	35.9	35.4	1.02	0.74	0.02	0.22
ADF, %	33.7	32.9	1.06	0.60	0.30	0.28

^a^*P* value associated with RFI, time, and the interaction of RFI and time

^b^*DM* dry matter, *CP* crude protein, *NDF* neutral detergent fiber, *ADF* acid detergent fiber

### Plasma variables

The results of the plasma variables are listed in Table 4. High RFI cows tended to have higher concentrations of triglycerides (*P* = 0.09) and ALT (*P* = 0.08) than those in low RFI cows. No differences were found in the other plasma variables (*P* > 0.10) between the two groups, with no interaction of day and RFI.

**Table 4:** Plasma variables of lactating cows selected for phenotypic divergence in residual feed intake (RFI)

Items^a^	Low RFI	High RFI	SEM	*P* value^b^		
				RFI	Day	RFI × Day
Protein metabolism						
Total protein, g/L	70.8	71.8	0.99	0.51	0.75	0.39
Albumin (A), g/L	26.7	27.6	0.41	0.12	0.78	0.54
Globulin (G), g/L	44.2	44.2	1.02	0.99	0.81	0.51
A/G	0.61	0.63	0.02	0.39	0.92	0.69
BUN, mmol/L	5.63	6.23	0.31	0.18	0.21	0.18
Creatinine, μmol/L	74.9	72.2	2.27	0.38	0.17	0.43
Energy substrates						
Glucose, mmol/L	3.61	3.48	0.09	0.35	0.42	0.61
NEFA, μmol/L	118	109	6.79	0.35	0.01	0.10
Triglyceride, μmol/L	0.17	0.28	0.04	0.09	0.75	0.95
Cholesterol, mmol/L	7.98	7.55	0.27	0.27	0.11	0.42
Liver function						
ALT, U/L	26.2	28.3	0.80	0.08	0.27	0.71
AST, U/L	91.4	88.7	5.00	0.71	0.43	0.85
ALP, U/L	34.6	47.0	5.14	0.11	0.13	0.26
Total bilirubin, μmol/L	1.56	1.49	0.06	0.43	0.49	0.64

^a^*BUN* blood urea nitrogen, *NEFA* nonesterified fatty acids, *ALT* alanine aminotransferase, *AST* aspartate aminotransferase, *ALP* alkaline phosphatase

^b^*P* value associated with RFI, time, and the interaction of RFI and time

### Microbial protein production

The results of the IADP, urinary PD, estimated MCP and MP are presented in Table 5. No differences were observed in the urinary PD (*P* = 0.86), estimated MCP *(P* = 0.86) and MP (*P* = 0.47). However, the amount of IADP tended to be greater in the high RFI group (*P* = 0.06) than that in the low RFI animals. The efficiency of rumen MCP synthesis was similar between the high and low RFI cows (*P* > 0.05). The low RFI cows had a greater proportion of dietary protein secreted into milk (*P* < 0.05) than that in the high RFI cows.

**Table 5:** The urinary purine derivatives (PD) and estimated MP supply to the dairy cows selected for phenotypic divergence in residual feed intake (RFI)

Items	Low RFI	High RFI	SEM	*P* value^a^		
				RFI	Day	RFI × Day
Urine volume^b^, L/d	33.6	33.4	2.64	0.96	< 0.01	0.07
Urinary PD, mmol/d						
Allantoin	489	520	33.1	0.51	0.60	0.16
Uric acid	39.4	51.1	4.80	0.11	0.09	0.40
Endogenous PD	54.2	49.9	2.87	0.31	0.29	0.23
Sum	474	484	40.2	0.86	0.70	0.77
MCP^c^, g/d	2152	2198	183	0.86	0.70	0.78
IADP^d^, g/d	695.5	760.8	22.5	0.06	0.07	0.80
MP^e^, g/d	2065	2171	100.2	0.47	0.36	0.58

^a^*P* value associated with RFI, time, and the interaction of RFI and time

^b^Urine volume (L/d) = BW (kg) × 29 (mg/d)/creatinine (mg/L) [24]

^c^Microbial protein (MCP) was indirectly estimated by the equation [21]: MCP = (allantoin + uric acid - endogenous PD) × 70 × 6.25/ (0.116 × 0.83 × 1000)

^d^Intestinally absorbable dietary protein (IADP) = RUP × CP intake × IDP, where IDP is the measured intestinal digestibility of rumen undegraded protein (RUP). The feedstuff incubated in the rumen for 16 h was used to determine the IDP according to a modified 3-step procedure [25]

^e^MP = IAMCP + IADP; IAMCP = Intestinally absorbable MCP = MCP × 0.64 [13]

### Partitioning of nitrogen

Nitrogen intake in high RFI cows tended to be greater than that in the low RFI cows (*P* = 0.06, Table 6). When expressed as a percentage of dietary N intake, N in milk was greater in low RFI (efficient) cows than in the high RFI cows (*P* = 0.02), but the N retained in the low RFI cows tended to be less than that in the high RFI cows (*P* = 0.06). However, the output of urine (*P* = 0.31) and feces (*P* = 0.91) and their proportion of dietary N (*P* > 0.05) were not different between the two groups.

**Table 6:** Nitrogen output and partitioning in lactating cows selected for phenotypic divergence in residual feed intake (RFI)

Items	Low RFI	High RFI	SEM	*P* value^a^		
				RFI	Day	RFI × Day
N intake, g/d	590	644	18.6	0.06	0.01	0.92
N output, g/d						
Feces	202	218	10.3	0.31	0.77	0.64
Urine	206	204	13.1	0.91	0.15	0.59
Milk	175	171	8.42	0.75	< 0.01	0.59
Retention^b^	5.72	51.4	14.1	0.04	0.15	0.82
% of N intake						
Feces	34.6	33.8	1.55	0.73	0.24	0.69
Urine	35.1	32.3	2.28	0.39	0.03	0.80
Milk	29.7	26.5	0.89	0.02	0.22	0.71
Retention	0.37	7.08	2.50	0.06	0.17	0.86

^a^*P* value associated with RFI, time, and the interaction of RFI and time

^b^Nitrogen retention = ingested N − fecal N − urinary N − milk N

## Discussion

With regard to the efficiency of N utilization in dairy cows, it is needed to consider the conversion of dietary CP into MP (due to differences in digestibility, ruminal fermentation, and absorption of nutrients, etc.) and the subsequent efficiency of MP conversion to milk protein. The MP is the milk protein precursor and its yield is closely related to milk and milk protein yield [13]. In our study, the increased tendency for IADP in the high RFI cows compared with that in the low RFI cows may be attributed to the greater DMI in these cows. However, the similar MCP may reduce the differences of the MP supply between two groups corresponding to the similar milk protein yield. Numerically, though not significantly, higher ratios of MCP to RDP and MP to CP intake were obtained in the low RFI cows compared to high RFI cows, eventually resulting in higher proportion of milk protein to the dietary CP. Our results are in line with Griffin et al. [26], who found that the low RFI animals were more efficient in their utilization of MP. Thus, the greater efficiency of each step for supply of MP may contribute to the higher percentage of N intake into milk protein, reflective of the greater efficiency in the low RFI cows. In the current study, we did not measure the rumen passage rate that may influence N efficiency; a relatively small sample size may not avoid between-animal variation (in creatinine excretion) in MCP estimation. Further work with a large cohort of animals is needed to validate the findings from the current study.

Richardson and Herd [27] suggested that digestion accounted for approximately 10% of the variation in RFI. Cantalapiedra-Hijar et al. [28] found a negative correlation between DM digestibility and DM intake and suggested that DM digestibility might be higher in low RFI cattle. Other study also reported that DM and CP digestibility is greater for low RFI dairy heifers fed fresh pasture [10] and for beef cattle [12]. However, nutrient digestibility were not different between the cows with high or low RFI, though high and low RFI cows had different DMI in our study, which is in agreement with some other findings [29–32]. These discrepancies among studies could be partially attributed to the differences in the types of diets fed and the methods used. In the study of Mauch et al. [33] with pigs and the study of Potts et al. [11] with dairy cows, the relationship between feed efficiency and digestibility was less significant for diets that are easier to be digested. Using internal markers (lignin, acid-insoluble ash and indigestible NDF), Cruz et al. [32], Lawrence et al. [31] and Potts et al. [11] all failed to found an association between diet digestibility and RFI. Systematic and random errors can increase when using internal markers, which may limit the ability to detect differences in digestibility.

Though low RFI cows consumed less DM, their total VFA concentrations and ruminal pH are similar to those in high RFI group, which is consistent with other results [10, 31, 34]. However, Guan et al. [35] reported that more efficient steers tended to have a greater concentration of total VFA and butyrate compared with less efficient cattle. In the current study, no differences were observed in the concentration of total VFA and molar proportion of the individual VFAs between the two groups. The ruminal VFA profile is the results of rumen epithelial absorption and microbial fermentation, but it does not directly reflect how the animal utilizes these products [36]. Therefore, more researches are needed to investigate the effects of RFI classification on the rumen microorganisms and the ability of the rumen epithelium to absorb and metabolize VFAs.

Triglycerides are stored in fat cells and serve as a source of energy [37]. Cameron [38] reported that plasma triglycerides are useful indicators of energy status in sheep. The low RFI cows had a lower concentration of plasma triglycerides than the high RFI cows, reflective of their lower milk fat content. Moreover, Phuong et al. [39] showed the strong correlation between energy and N metabolism. Thus, different triglycerides indicated the different energy status between two groups and may affect N metabolism. Sakowski et al. [40] reported that increased ALT activity can be associated with the risk in liver disorders that are commonly caused by negative energy balance during early lactation stage. Thus, the tendency for higher ALT in high RFI cows indicates the higher health risk in these cows.

The total amounts of N partitioned to milk, feces and urine were not different between the two RFI cows, which agrees with the findings of Lines et al. [41]. However, Marett et al. [29] reported a reduction in urinary N excretion for lower RFI cow. In their study, Marett et al. [29] selected primiparous and multiparous cows based on RFI measured in calves, and neglect of parity may lead to naive assessment of the effect of RFI on nitrogen portioning. In contrast to the greater DMI by high RFI cows, cows with low RFI partitioned a greater percentage of dietary N into milk protein in our study. In disagreement with our study, Rius et al. [10] found no difference in N partitioning in milk, but cows with low RFI had lower N output in milk and feces and tended to have lower milk yield, compared with those of the high RFI. The conflicting relationships between N partitioning and RFI among studies could partially account for differences in milk yield. Rius et al. [10] demonstrated a trend for lower milk yield in low RFI cows than that of high RFI cows. Conversely, the milk yield of animals for two groups was similar in our study. Although the partitioning of N in feces and urine was not significantly different between the RFI phenotypes, a greater value from the low RFI cows would have been expected in terms of their higher MUN concentrations, compared with cows with high RFI. In the present study, the low RFI cows retained less N than the high RFI cows, which may be due to their lower DMI. Moreover, other studies with male cattle [42] and young bulls [43] have proposed that N retention increases due to a rise in the N supply. However, the ADG of two groups were not different, which is inconsistent to the differences in N retention. Lines et al. [41] found that the divergence in RFI is attributed to the differences of fat deposition, with extra deposition of energy as fat in high-RFI cattle with extra feed (energy) intake. Thus, high milk-protein-yielding cows selected for low RFI partitioned a greater percentage of N intake into milk protein, which may provide the dairy farm with more economic profit.

## Conclusion

The present experiment highlights the limited variation in N utilization for milk production in cows producing high milk protein yields with different RFI. Several metabolic and physiological processes, including ruminal fermentation and N partitioning, may potentially contribute to these results. Selection for low RFI may lead to a greater proportion of N to milk and less to retention, which may improve the economic benefits of dairy farmers. While this study mainly focused on N metabolism, energy partitioning may also contribute to the variation in RFI. Thus, further research is required to better understand whether or how energy partitioning is responsible for variation in RFI in high milk protein yield dairy cows.

## Additional file

Additional file 1: **Table S1.** Degradation constants of dry matter (DM) and crude protein (CP) based on the eq. *P* = *a* + *b*[1 − exp(−*c*t)], where *P* = the rate of disappearance at time *t* (h), *a* = the rapidly degradable fraction in the rumen, and *b* = the fraction slowly degraded at rate *c* (*c* > 0); their effective degradability (dg); and rumen undegraded protein (RUP) of the experimental diet. (DOCX 22 kb)

## Abbreviations

ADF: Acid detergent fiber
ADG: Average daily gain
ALP: Alkaline phosphatase
ALT: Alanine aminotransferase
AST: Aspartate aminotransferase
BUN: Blood urea nitrogen
BW: Body weight
CP: Crude protein
DGGS: Distillers dried grains with soluble
DM: Dry matter
DMI: Dry matter intake
ECM: Energy-corrected milk yield
IADP: Intestinally absorbable dietary protein
IDP: Intestinal digestibility of RUP
iNDF: Indigestible neutral detergent fiber
MCP: Microbial protein
MP: Metabolizable protein
MUN: Milk urea nitrogen
N: Nitrogen
NDF: Neutral detergent fiber
NEFA: Non-esterified fatty acids
PD: Purine derivatives
RDP: Rumen degradable protein
RFI: Residual feed intake
RUP: Rumen undegradable protein
SCC: Somatic cell count
TS: Total solids
VFA: Volatile fatty acids
