# Supplementary material for: Nitrogen partitioning and microbial protein synthesis in lactating dairy cows with different phenotypic residual feed intake
Source: J Anim Sci Biotechnol. 2019 Jun 14;10:54. doi: 10.1186/s40104-019-0356-3 (PMC6580507; doi:10.1186/s40104-019-0356-3)
Supplement: Supplementary file 1 — Table S1. Degradation constants of dry matter (DM) and crude protein (CP) based on the eq. P = a + b[1 − exp(−ct)], where P = the rate of disappearance at time t (h), a = the rapidly degradable fraction in the rumen, and b = the fraction slowly degraded at rate c (c > 0); their effective degradability (dg); and rumen undegraded protein (RUP) of the experimental diet. (DOCX 22 kb) [file 40104_2019_356_MOESM1_ESM.docx]

**Supplemental material**

**Table S1.** Degradation constants of dry matter (DM) and crude protein (CP) based on the equation *P* = *a* + *b*[1 − exp(−*c*t)], where *P* = the rate of disappearance at time *t* (h), *a* = the rapidly degradable fraction in the rumen, and *b* = the fraction slowly degraded at rate *c* (*c* >0); their effective degradability (dg); and rumen undegraded protein (RUP) of the experimental diet

| Items | Mean | | SEM |
| --- | --- | --- | --- |
| DM degradation | |  | |
| *a*, % | 26.25 | | 1.09 |
| *b*, % | 52.21 | | 2.01 |
| *c*, %/h | 8.01 | | 0.40 |
| dg^a^ | 59.44 | | 1.63 |
| CP degradation | |  | |
| *a*, % | 24.23 | | 1.61 |
| *b*, % | 69.08 | | 1.80 |
| *c*, %/h | 7.95 | | 0.66 |
| dg^a^ | 67.92 | | 0.80 |
| RUP^b^, % of CP | 32.08 | | 0.80 |

^a^dg = *a* + *bc*/(*c* + kp) [1] assuming a passage rate (kp) of 4.6%/h [2].

^b^RUP = 100 − RDP.

**References**

1. Orskov ER, Hovell DDD, Mould F. The use of the nylon bag technique for the evaluation of feedstuffs. Trop Anim Prod. 1980; 5: 195-213.
2. Krizsan SJ, Ahvenjärvi S, Huhtanen P. A meta-analysis of passage rate estimated by rumen evacuation with cattle and evaluation of passage rate prediction models. J Dairy Sci. 2010; 93:5890-901.
